# Spatial Relational Memory Requires Hippocampal Adult Neurogenesis

**DOI:** 10.1371/journal.pone.0001959

**Published:** 2008-04-09

**Authors:** David Dupret, Jean-Michel Revest, Muriel Koehl, François Ichas, Francesca De Giorgi, Pierre Costet, Djoher Nora Abrous, Pier Vincenzo Piazza

**Affiliations:** 1 INSERM U862, Institut F. Magendie, Bordeaux, France; 2 University of Bordeaux 2, Bordeaux, France; 3 INSERM E347, Institut Bergonié, University of Bordeaux 2, Bordeaux, France; 4 Laboratoire de Transgénèse, University of Bordeaux 2, Bordeaux, France; Columbia University, United States of America

## Abstract

The dentate gyrus of the hippocampus is one of the few regions of the mammalian brain where new neurons are generated throughout adulthood. This adult neurogenesis has been proposed as a novel mechanism that mediates spatial memory. However, data showing a causal relationship between neurogenesis and spatial memory are controversial. Here, we developed an inducible transgenic strategy allowing specific ablation of adult-born hippocampal neurons. This resulted in an impairment of spatial relational memory, which supports a capacity for flexible, inferential memory expression. In contrast, less complex forms of spatial knowledge were unaltered. These findings demonstrate that adult-born neurons are necessary for complex forms of hippocampus-mediated learning.

## Introduction

For many years, it was believed that the adult mammalian brain is composed of a fixed number of neurons that no longer divide after the end of development. However, in very restricted brain areas, new neurons are produced throughout the lifespan of individuals [1]. This discovery led to the fascinating but still controversial hypothesis that the encoding of specific information requires the integration of adult-born neurons in existing brain circuits. The dentate gyrus (DG) of the hippocampus is one of these few regions of the mammalian brain where new neurons are generated throughout adulthood [1]. These adult-born granule neurons are integrated into the hippocampal circuitry and exhibit electrophysiological properties similar to those of mature granule neurons [2].

The DG is part of an integrated network that plays an important role in memory processes and in particular in the establishment and use of spatial representations [3], [4]. Spatial memory is classically assessed in the water maze since this hippocampal-dependent task, which requires the animal to navigate through space, depends on the encoding and flexible use of positional relationships between cues [3]. Based on several correlative pieces of evidence it was initially hypothesized that adult-born hippocampal neurons are involved in spatial memory assessed in this water maze. For example, conditions that improve learning abilities in the water maze, such as enriched environments or middle-age adrenalectomy, also enhance neurogenesis [5], [6]. Conversely, conditions that impair spatial learning, such as prenatal stress or lesions of the cholinergic septo-hippocampal pathway, decrease neurogenesis [7], [8]. In addition, a positive correlation between neurogenesis and learning performance was found in senescent rats [9]. Finally, spatial learning modifies the production and survival of newborn neurons [10]–[15].

Despite these compelling correlative pieces of evidence, more recent attempts to demonstrate a causal relationship between neurogenesis and spatial learning have generated controversial results (for review see [16], [17]). Indeed, after ablating neurogenesis, spatial memory deficits have been reported in some studies [18], [19] but not in others [20]–[25].

Two major factors could explain this discrepancy. First, the approaches used to block neurogenesis in the adult brain have up to now lacked specificity. Indeed, these methods, which include high energy irradiation or treatment with the DNA methylating agent methylazoxymethanol acetate (MAM), lack selectivity and have unwanted side effects [26]–[28]. Second, the behavioral protocols used in various studies may present variable complexities that require the involvement of the hippocampus to different degrees.

The functional role of neurogenesis thus remains an open question. To address this issue, we first developed a new tool to impair neurogenesis. More specifically, we generated transgenic mice in which the death of neural precursors can be selectively induced by over-expressing the pro-apoptotic protein Bax. Then, we studied these mice using spatial tasks of different complexities that involve different degrees of hippocampal dependency. We found that specific ablation of adult-born hippocampal neurons leads to an impairment of spatial relational memory whereas simpler forms of spatial knowledge remain unchanged. These findings demonstrate that adult-born granule neurons are involved in hippocampo-dependent spatial memory depending on the cognitive demand.

## Results

### Development of an inducible and cell-specific transgenic strategy

**Figure 1:**
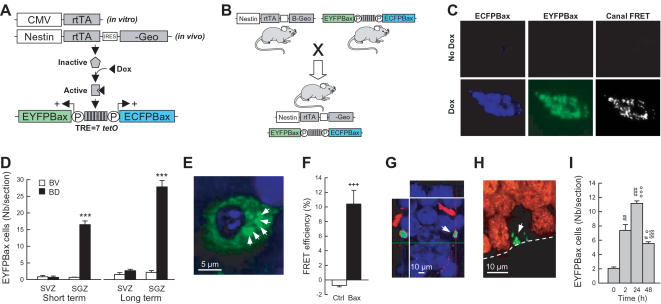
Inducible expression of Bax proteins in hippocampal neural precursors using a Tet-On system. (A) Schematic representation of the inducible Tet-ON system. rtTA expression is driven either *in vitro* by the CMV promoter in stably transfected CHO-K1 Tet-ON cells or *in vivo* by the rat nestin intron II enhancer/promoter. Doxycycline (Dox) activates the rtTA protein, which binds to seven TetO sequences (TRE) to drive the co-transcription of ECFPBax and EYFPBax transgenes. (B) Strategy to obtain Dox-dependent ablation of neural precursors *in vivo* using double transgenic nestin-rtTA/Tet-Bax mice. (C) Six hours of Dox treatment induces ECFPBax and EYFPBax expression and multimerization in CHO-K1 Tet-ON cells. The presence of ECFPBax/EYFPBax multimers at the mitochondrial membrane was confirmed by FRET analysis. (D) Number of Bax multimers in the subventricular zone (SVZ) and the subgranular zone (SGZ) of bigenic mice treated with (bigenic-Dox mice, BD) or without (bigenic-vehicle mice, BV) Dox (2 mg/ml) during a short term (3 weeks) or a long term (17 weeks) period. (E) Confocal illustration showing EYFPBax (green) clusters (arrows) in the cytoplasm of cells located in the SGZ of bigenic-Dox mice (blue = hoescht nuclear counterstaining). (F) FRET analysis of Bax multimers shows the intermolecular interaction between ECFPBax and EYFPBax fusion proteins. FRET efficiency measured following EYFPBax acceptor photobleaching results in a brightening of the ECFPBax donor fluorescence in areas devoid of (Ctrl) or containing (Bax) Bax multimers. (G) Confocal illustration showing that nestin-IR neuronal precursors (red) are positive for EYFPBax (green). Hoescht nuclear counterstaining is shown in blue. (H) Confocal illustration showing that NeuN-IR mature granule neurons (red) are negative for EYFPBax (green). (I) Number of Bax multimers in the SGZ of bigenic mice treated acutely with Dox (2 mg/ml/100 g body weight, i.p.) and sacrificed at different time intervals. ***: p≤0.001 compared to BV, ^+++^: p≤0.001 compared to Ctrl areas, ^#^: p≤0.05, ^##^: p≤0.01, ^###^: p≤0.001 compared to 0 h, ^°^: p≤0.05, ^°°°^: p≤0.001 compared to 2 h, ^§§§^: p≤0.001 compared to 24 h.

To demonstrate a causal relationship between adult neurogenesis and spatial learning, we generated double transgenic mice (referred as “bigenic mice”) in which neural precursors can be selectively killed by over-expressing the pro-apoptotic Bax protein (Figures 1A & 1B), a key protein that mediates the death of newborn cells [29], [30]. In response to apoptotic stimuli or when artificially over-expressed, Bax multimerizes at the outer mitochondrial membrane, inducing the release of cytochrome *c*, which triggers the apoptotic cascade leading to cell death [31]. The inducible over-expression of Bax specifically in neural precursors was obtained using the reverse tetracycline-controlled transactivator (rtTA)-regulated system [32]. rtTA is a transcription factor that recognizes specific response elements (Tetracycline Response Element, TRE) in the promoter region of target genes. It can be expressed under the control of cell-specific promoters and activated by the administration of an exogenous tetracycline analogue, doxycycline (Dox).

The transgenic mice we developed (referred as “Bax mice”, Figure 1B) integrated a bidirectional construct in which a TRE responsive minimal CMV (CytoMegaloVirus) promoter drives the expression of two Bax proteins fused with different green fluorescent protein spectral mutants (ECFPBax & EYFPBax, Figures 1A & 1B). As shown by the *in vitro* testing of this construct (Figure 1C), the fusion of Bax with fluorescent proteins did not impair its pro-apoptotic properties and allowed the control of both the expression of the transgene and its multimerization by using Fluorescence Resonance Energy Transfer (FRET) [33].

Bax mice were then crossed with regulatory mice in which the rtTA transgene was controlled by the rat nestin intron II enhancer/promoter [34] which is specifically expressed in neural precursors [35] (Figure 1B). These regulatory mice were previously shown to express the rtTA transgene selectively in the developing brain [34]. Yet, no published data exist on the expression of this transgene in the adult brain. However it is specified on the Jackson laboratory's website that the rtTA transgene can be expressed in the cerebellum, and in the major neurogenic zones of the adult brain thus including the DG and the subventricular zone (SVZ). Because of the lack of precise information in the adult brain, we characterized transgene expression in our rtTA-Bax bigenic mice.

### Doxycycline treatment in bigenic rtTA-Bax mice reduces hippocampal neurogenesis

#### Influence of Dox treatment on Bax expression

In bigenic mice containing both Tet-Bax and nestin-rtTA constructs, a short-term Dox treatment (three weeks, 2 mg/ml) induced the expression of EYFPBax proteins in the DG (Figure 1D, t_5_ = −12.1, p<0.001) but not in the SVZ (Figure 1D, t_5_ = 0.28, p>0.78). Bax fusion proteins formed cytoplasmic and perinuclear clusters (Figure 1E) restricted to the subgranular zone (SGZ) of the DG where neural precursors reside. As shown by FRET analysis, these clusters were constituted by EYFPBax and ECFPBax protein multimers (Figure 1F, t_11_ = −7.57, p<0.001). Confocal analysis revealed that EYFPBax proteins were present in nestin expressing cells (Figure 1G) but never in NeuN expressing mature granule neurons (Figure 1H). Doxycycline treatment induced the expression of Bax clusters in an inducible and reversible way. Thus, when Dox (2 mg/ml) was acutely administered by i.p. injection, an increase in EYFPBax proteins was observed 2 hours after treatment, reaching peak expression 24 hours later, and tended to go back to basal levels 48 hours later (Figure 1I, time effect: F_3,11_ = 33.23, p<0.001). We then determined the effect of a long-term Dox treatment (17 weeks, 2 mg/ml) in another batch of animals that were behaviorally tested (see below). We found, as shown for a 3-weeks Dox treatment, a significant increase in the expression of EYFPBax fusion protein in the DG (Figure 1D, t_10_ = −13.1, p<0.001) but not in the SVZ (Figure 1D, t_10_ = −1.66, p>0.12). In these animals, we also analyzed the expression of the EYFPBax fusion protein in several brain areas. No increase in EYFPBax expression was found in the olfactory bulb, the cerebellum, the striatum and the frontal cortex (Supplementary Table S1 and Figure S1).

#### Influence of Dox treatment on neurogenesis

**Figure 2:**
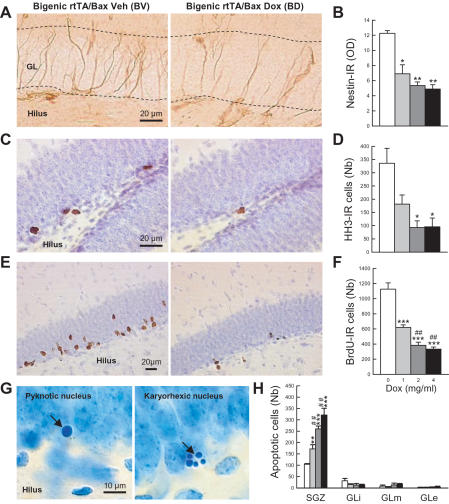
Effects of short-term Dox treatment on adult hippocampal neurogenesis. (A) Illustration of nestin staining. (B) Densitometric analysis of nestin-IR. (C) Illustration of dividing cells visualized by HH3 staining. (D) Number of HH3-IR cells. (E) Illustrations of newborn cells visualized by BrdU staining. (F) Number of BrdU-IR cells. (G) Illustrations of apoptotic cells. (H) Number of apoptotic cells in the subgranular zone where newborn cells are produced (SGZ) and in the internal (GLi), medial (GLm) and external (GLe) granular cell layer. GL = granular cell layer. *: p≤0.05, **: p≤0.01, ***: p≤0.001 in comparison to the bigenic-vehicle group. ##: p≤0.01, in comparison to bigenic fed with Dox at 1 mg/ml.

After a short-term Dox treatment (3 weeks), the over-expression and subsequent multimerization of Bax proteins in bigenic rtTA-Bax mice induced death of neural precursors as shown by three dose-dependent effects of Dox treatment (0, 1, 2, 4 mg/ml). First, a reduction of nestin immunostaining was observed following Bax over-expression (Figures 2A & 2B; group effect: F_3,11_ = 6.29, p<0.009). This result was expected since nestin-expressing cells are targeted by our transgenic construct (Figures 1B & 1G). Second, we observed a profound decrease in cell proliferation as measured by staining for the phosphorylated Histone H3 (HH3, Figures 2C & 2D; group effect: F_3,11_ = 4.01, p<0.04). Third, the number of 12-day-old newborn cells labeled with the thymidine analogue BrdU was also reduced (Figures 2E & 2F; group effect: F_3,11_ = 33.99, p<0.001). When analyzing the effects of Dox treatment along the rostro-caudal axis of the DG, we found that this treatment decreased the number of BrdU-labeled cells throughout the DG (Supplementary Figure S2). Finally, a specific increase in cell death was visualized in the SGZ where newborn cells reside (Figures 2G & 2H, SGZ, group effect: F_3,11_ = 23.39, p<0.001) but not in the internal (GLi, group effect: F_3,11_ = 0.55, p>0.66), medial (GLm, group effect: F_3,11_ = 0.73, p>0.55) and external (GLe, group effect: F_3,11_ = 0.20, p>0.89) parts of the granule cell layer (GL) constituted in majority by mature neurons. In contrast to what was observed in the SGZ of the DG, cell genesis was not significantly modified after a 3-week treatment of Dox in the SVZ (Supplementary Table S2), the second main neurogenic area of the adult brain [1]. This finding is consistent with the lack of Dox-induced increase in the expression of EYFPBax fusion proteins in this brain area (Figure 1D).

**Figure 3:**
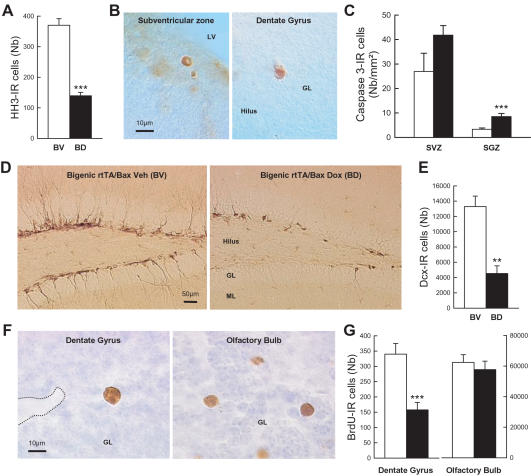
Effects of long-term Dox treatment on adult neurogenesis. (A) Number of HH3-IR cells in the dentate gyrus (DG) of bigenic mice treated with (BD, black) or without (BV, white) Dox. (B) Illustration of Caspase 3-IR cells in the subventricular zone (SVZ) and the subgranular zone (SGZ) of the DG. (C) Density of Caspase 3-IR cells in the SVZ and the SGZ of bigenic (black) and control mice (white) treated with Dox. (D) Illustrations of immature neurons visualized by Dcx staining in the DG. (E) Number of Dcx-IR immature neurons in the DG of bigenic mice treated with (black) or without (white) Dox. (F) Number of one-month-old BrdU-IR cells in the DG and the OB of bigenic (black) and control mice (white) treated with Dox. **: p≤0.01 and ***: p≤0.001 compared to control groups.

We further characterized our model by studying the effects of a long-term Dox treatment (2 mg/ml of Dox for 17 or 19 weeks) on neurogenesis. In these conditions we found that the number of dividing cells expressing HH3 (Figure 3A; t_10_ = 9.52, p<0.001) was still decreased in the DG of bigenic-Dox mice. An increase in cell death, evaluated by the use of a specific apoptotic marker, the active-form of caspase 3 [14] was also observed in the SGZ (Figures 3B & 3C, t_13_ = −4.24, p<0.001) but not in other brain areas (Figures 3B & 3C, SVZ, t_13_ = −1.83, p = 0.09; & Supplementary Table S1). In order to verify that neurogenesis was reduced, doublecortine (Dcx) was used as a marker of newly born neurons [36], [37]. The number of newborn neurons expressing Dcx was decreased in the DG of bigenic-Dox mice (Figures 3D & 3E; t_10_ = 3.92, p<0.003). These alterations were associated neither with changes in the total number of granule cells nor with changes in the volume of the granule cell layer (Supplementary Table S3). Furthermore, cell proliferation and the number of new neurons did not differ among the different control groups (Supplementary Figures S3A & S3B).

To verify if the survival of the remaining newborn neurons, i.e. those unaffected by Bax over-expression, was increased as a compensatory response to the lower number of newborn cells, animals of the last experiment received a BrdU pulse four weeks before sacrifice. It was found that in this condition the number of BrdU-labeled cells was also decreased in the DG of bigenic mice treated with Dox (Figures 3F & 3G, t_13_ = 4.37, p<0.001). Since newborn cells produced in the SVZ migrate to the olfactory bulb (OB) we also evaluated the number of 4-week-old BrdU-labeled cells in this structure. No differences in the number of BrdU-labeled cells between control and bigenic animals treated with Dox (Figure 3G, t_13_ = 0.97, p>0.34) were found. The volume of the OB was similar in the two groups (CD: 2.64±0.15 mm^3^, BD: 2.40±0.08 mm^3^, t_13_ = 1.43, p>0.17). This is consistent with the lack of Dox-induced increased expression of EYFPBax fusion proteins in the SVZ (Figure 1D).

Finally, we analyzed the potential collateral consequences of ablating new neurons in inflammatory and vascular responses in the DG. Staining for the platelet endothelial cell adhesion molecule-1 (PECAM-1-IR), which labels blood vessels, did not show differences between control and bigenic mice treated with Dox (Supplementary Figures S4A & S4B, t_13_ = −0.39, p>0.70). Similarly the two groups did not differ in the number of cells stained for the complement type 3 receptor CD11b which labels microglia (Supplementary Figures S4C & S4D, t_13_ = 0.36, p>0.71).

In conclusion, these observations confirm that in bigenic mice Dox treatment induces the over-expression of Bax transgenes in the DG leading to a specific inhibition of hippocampal neurogenesis.

### Inhibition of adult hippocampal neurogenesis alters spatial relational memory

We then characterized these transgenic mice behaviorally to investigate the role of adult neurogenesis in hippocampal-dependent spatial learning. The hippocampus has been implicated to different degrees for a broad range of behavioral processes involving increasing complexities of learning and memory of space, such as detection of novel environments, contextual conditioning and spatial navigation [38], [39]. We analyzed the role of neurogenesis in these different forms of spatial learning by comparing adult bigenic mice receiving Dox (2 mg/ml for 6 weeks prior to behavior) with the appropriate controls.

**Figure 4:**
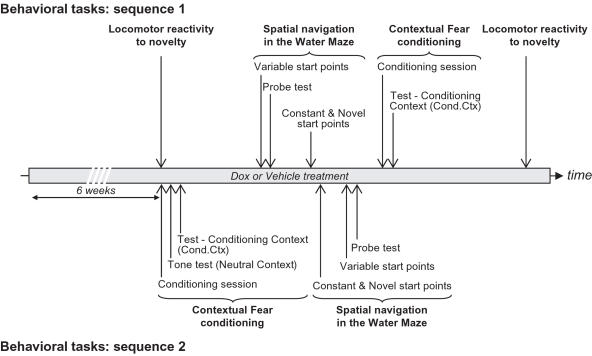
Diagram of behavioral task sequences.

All behavioral experiments were performed twice in two independent experiments in which the sequence of the behavioral tasks was inverted (Figure 4). These experiments showed that the order of the task did not influence the behavioral consequences of neurogenesis inhibition. Thus, we present here for each task only the results of the experiment in which the task was performed first in the experimental sequence (see the complete set of results in Supplementary Figure S5).

In order to verify for non-specific effects of genetic and pharmacological manipulations, the complete range of mice strains (bigenic mice, wild-type mice, and the two parental strains: Tet-Bax and nestin-rtTA mice) treated or not treated with Dox were studied in experiment 2. It was found that none of the control groups (wild-type, Tet-Bax and nestin-rtTA mice) treated either with vehicle or Dox differed from bigenic mice treated with vehicle (Supplementary Figures S3C–F). For this reason, in experiment 3 all mice were treated with Dox and bigenic mice were compared to the control strains (wild-type, Tet-Bax and nestin-rtTA mice).

**Figure 5:**
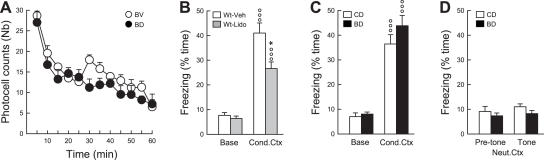
Effects of adult hippocampal neurogenesis ablation on simple forms of spatial knowledge. (A) Exploration in a novel environment measured by photocell beam breaks. (B) Contextual fear conditioning assessed by the percentage of freezing displayed by C57BL/6J wild-type (Wt) mice before conditioning (Base) and when re-exposed 24 hours later to the conditioning context (Cond.Ctx). Mice were infused with Vehicle (Veh) or lidocaine (Lido) in the dorsal hippocampus before conditioning. (C) Contextual fear conditioning assessed by the percentage of freezing displayed by control (CD) and bigenic mice (BD) treated with doxycycline in the basal condition and when re-exposed to the conditioning context 24 hours later. (D) Fear conditioning assessed by the percentage of freezing displayed 24 hours after conditioning by CD and BD mice when exposed to a neutral context (Neut.Ctx) before (Pre-tone) and during (Tone) re-exposure to the tone present during conditioning but unpaired with the shock. BV = bigenic-vehicle mice. °°°: p≤0.001 compared to Base. *: p≤0.05 compared to Wt-Veh.

We first analyzed the influence of depleted neurogenesis on habituation to a novel environment, which is considered a simple form of spatial recognition measured by a decrease over time in exploratory activity as the context loses its novelty. Hippocampal dysfunction impairs this form of spatial recognition and delays habituation, which results in increased locomotor activity in the novel environment [38]. In this task, bigenic-Dox mice were not impaired showing similar novelty-induced locomotor activity than bigenic-vehicle mice (Figure 5A; group effect: F_1,10_ = 1.25, p>0.28; interaction group×time: F_11,110_ = 0.71, p>0.72).

Contextual fear conditioning requires learning and remembering an association between environmental cues and an electric shock. If learning occurs, further exposure of the animal to the conditioning environment elicits a freezing fear response. The protocol used in our experiments, by systematically unpairing a discrete auditory cue (a tone) with the shock, facilitates the conditioning to the context [40]. Performances in this task are impaired by disruption of hippocampus function. Thus, inactivation of the dorsal hippocampus by lidocaine injection reduced contextual freezing by approximately 35% (Figure 5B, Veh *vs* Lido: t_11_ = 2.65, p<0.05) and this deficit did not exceed 65% even after a complete lesion of the hippocampus [40]. In this condition, inhibition of neurogenesis did not have significant effects on contextual fear conditioning as shown by the lack of difference in contextual freezing between control-Dox and bigenic-Dox mice (Figure 5C, t_13_ = −1.26, p>0.23). The conditioning obtained in this experiment was specific to context since no increase in freezing was observed after the exposure of animals to a neutral context (Figures 5C & 5D, Base *vs* Pre-tone: t_12_ = 0.04, p>0.96) or to the auditory-cue which was present during conditioning but was not associated with shock occurrence (unpaired tone, Figure 5D, Pre-tone *vs* Tone: t_12_ = −0.32, p>0.75).

Spatial navigation was studied using the water maze in which animals learn the location of a hidden platform using distal cues. This task can be solved using multiple strategies in parallel, which requires the integrity of the hippocampus to different degrees [41], [42]. The strategies that the animals develop depend on the type of training that is used. In our experiments we used two training procedures that allow the development of hippocampal-dependent and hippocampal-independent learning, respectively.

**Figure 6:**
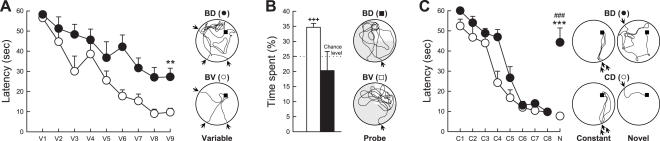
Effects of adult hippocampal neurogenesis ablation on spatial navigation. (A) Latency to reach the hidden platform using variable start positions. Right: representative swim paths during the last training day (V9) for a bigenic mouse treated with Dox (BD) and a bigenic mouse treated with vehicle (BV). (B) Time spent in the target quadrant during the probe test. Right: representative swim paths during the probe test. (C) Latency to reach the hidden platform using constant (C1 to C8) or novel (N) start positions. Right: representative swim paths during constant (C8) and novel (N) start position training days. BV = bigenic-vehicle mice; BD = bigenic-Dox mice; CD = control-Dox mice. ###: p≤0.001 compared to C8; **: p≤0.01, ***: p≤0.001 compared to the control group; +++: p≤0.001 compared to chance level. Black arrows indicate start point positions.

In the first hippocampal-dependent procedure (Figures 6A & 6B), from the beginning of the training phase the platform was maintained hidden, and the starting point was changed at each of the three daily trials. In this case, in order to find the hidden platform, the animal has to use an allocentric mapping strategy that consists of learning the positional relationships among multiple independent environmental cues (“spatial relational memory”). This relational representation is needed to support the flexible use of learned discriminative cues in novel situations (i.e. changing starting position), and is consequently necessary to solve the task. This cognitive ability is one of the most well-studied hippocampus-dependent abilities, since this task cannot be learned if the hippocampus is impaired [43], [44].

In the second hippocampal-independent procedure (Figure 6C) the platform was also hidden from the beginning of training but the starting point was maintained constant for all trials. In this case, although the development of a mapping strategy is not prevented, the animal can also learn the position of the platform using egocentric strategies consisting of, for example, an association of an invariant configuration of spatial cues to the escape platform (“place learning”). Egocentric strategies are very efficient for finding the platform if the starting point is maintained constant but fail to sustain the behavior if the starting point is suddenly changed. Egocentric strategies are hippocampus-independent and thus, if the starting point is maintained constant, animals can solve the water maze task even after a complete impairment of the hippocampus [44].

Inhibition of neurogenesis impaired spatial relational memory. Indeed, when submitted to the variable starting point procedure, bigenic-Dox mice were profoundly impaired in learning the platform location (Figure 6A; group effect: F_1,10_ = 16.33, p<0.002; group×time interaction: F_8,80_ = 2.80, p<0.008). This deficit was not due to a “non-cognitive strategy” such as passive floating as indicated by a positive correlation between latency and swim path length (Supplementary Table S4A). The memory deficit was confirmed by a probe test performed at the end of the learning sequence. For this test the platform is removed and the time spent by the animal in the area previously containing the platform (target quadrant) is measured. As classically observed, bigenic animals treated with vehicle spent more time in the quadrant previously containing the platform than in the other quadrants (Figure 6B, t_5_ = 8.92, p<0.001 against chance level). In contrast bigenic mice treated with Dox did not show any preference for the platform-associated quadrant (Figure 6B, t_5_ = −0.50, p>0.63 against chance level). This deficit observed in bigenic-Dox mice was neither due to sensory-motor deficits or thygmotaxic behavior (Supplementary Table S4B) nor to changes in visual acuity since these mice were able to locate a visible platform at the end of the testing period (Supplementary Table S4C).

In contrast, inhibition of neurogenesis did not influence learning when the hippocampal-independent procedure was used. Thus, when animals were trained with a constant starting point, bigenic-Dox mice were not impaired and learned the location of the hidden platform as well as control animals treated with Dox (Figure 6C; group×time interaction: F_7,91_ = 1.41, p>0.21; analysis on C8, t_13_ = −0.55, p>0.58). However, when the animals were tested from a new starting point at the end of the learning phase, bigenic-Dox mice were unable to find the platform despite previous learning of the platform location (Figure 6C; t_14_ = −6.17, p<0.001 compared to C8; t_13_ = −6.24, p<0.001 compared to CD). In contrast, control-Dox mice were very efficient in finding the platform from this novel start position (Figure 6C; t_14_ = 0.72, p>0.48, N compared to C8). This finding indicates that in parallel to egocentric strategies, the control animals also developed a relational strategy which allowed them to find the platform when the starting point was changed. In contrast, bigenic animals treated with Dox only developed egocentric strategies that proved inefficient for finding the hidden platform once the starting point was changed.

These results show that in the water maze, inhibition of neurogenesis in the DG of the hippocampal formation selectively impairs spatial relational memory while other forms of spatial navigation are spared. Taken together, the results of the behavioral experiments show that neurogenesis influences forms of learning requiring critical hippocampal involvement such as relational processing of spatial information. In contrast, other forms of learning that do not require such a process, and thus depend on the hippocampus to a lesser degree, such as novelty exploration or contextual fear conditioning, are not affected by inhibition of hippocampal neurogenesis.

## Discussion

### A novel inducible transgenic strategy for the ablation of nestin precursors

In this report we describe an original conditional transgenic approach that allows the ablation of nestin precursors in adult animals. This model is based on the inducible and cell type-restricted over-expression of the pro-apoptotic protein Bax using the reverse tetracycline-controlled transactivator (rtTA)-regulated system (Tet-On system) [32]. The Tet-On system (rtTA), which is not naturally present in mammalian cells, offers the best temporal/spatial flexibility and selectivity since it is inactive in its default state (i.e. absence of exogenous Dox). Indeed, the transgenes are kept inactive and are transcriptionally active only in the presence of Dox. The rtTA protein is under the control of the rat nestin intron II enhancer/promoter which is specifically expressed in neural precursors [45]–[47]. This is the reason why this promoter was preferred over a GFAP promoter [48], [49]. Furthermore, to ensure a narrow profile of tissue-specific promoter transcriptional activity we designed a bidirectional vector that first minimizes the genomic influence of cis-acting elements with enhancer/promoter activities, and secondly allows coordinate expression of two independent transgenes (EYFPBax and ECFPBax) from one TRE. Finally, by using the Bax protein, we took advantage of the constitutive molecular apoptotic pathway regulating natural cell death of adult-born granule neurons [30].

Activation of transgenes with Dox treatment resulted in the overexpression of Bax proteins in nestin expressing cells in the subgranular zone of the DG where neural precursors reside. This resulted in the death of neural precursors as indicated by a reduction in nestin staining and an increase in the number of apoptotic cells in the SGZ. Consequently cell proliferation and neurogenesis were reduced, albeit not totally blocked in the DG. Indeed, a residual level of neurogenesis persisted several months after the beginning of the Dox treatment. This is in line with some studies in which hippocampal neurogenesis was strongly though not fully blocked by irradiation [18], [20], [25], although others reported a total and persistent ablation of neurogenesis using the same technique [19], [21].

Expression of Bax fusion proteins, activation of caspase-3, as well as cell genesis and neurogenesis were not modified by Dox treatment in the SVZ-OB system, the second main neurogenic area of the adult brain. Furthermore, in other brain areas, such as the cerebellum, the cortex or the striatum neither the expression of Bax fusion proteins nor the number of cells expressing the activated form of caspase-3 were modified. Our data then show that in the adult brain, rtTA expression is more restricted than what is observed in the developing brain [34]. In addition to developmental differences, differences in mice strains could also be involved. Thus we maintained the transgenic line on a C57BL/6J background while Mitsuhashi et al. study was performed on mice from an FVB/N background. Finally it is well known that the pattern of expression in bigenic mice is often different from the one of the parental strains. This phenomenon is explained by the fact that transgenic methods generate embryos expressing transgenes in mosaic patterns due to differences in the integration sites, number of transgene copies and DNA methylation [50].

### Adult-born neurons influence spatial relational memory

The hippocampus has been implicated to different degrees in various forms of spatial knowledge involving increasing levels of complexity ranging from spatial recognition to contextual conditioning up to spatial relational memory [38], [39]. Our results show that neurogenesis is necessary for the expression of the most hippocampal-demanding form of spatial knowledge studied here: spatial relational memory. Indeed, the reduction of adult-born hippocampal neurons does not modify the ability of animals to habituate to a novel environment or to remember an environment associated with a punishment. In contrast, the acquisition of spatial relational memory, which requires the encoding and the flexible use of the positional relations between cues, is impaired by the inhibition of neurogenesis.

Novelty detection, contextual fear conditioning, place learning and spatial navigation not only differ quantitatively in terms of hippocampal dependency but also qualitatively in the way the learned information is used. In the relational version of the water maze, animals must encode geometric relationships among salient cues and use the relational properties of these distal cues to locate the goal from multiple views of the environment [3]. This relational encoding of the distal cues allows the use of previously learned information to infer problem-solving strategies in novel situations. In contrast, in contextual fear conditioning or during novelty detection, the environmental cues can be encoded, fused together and stored as a snapshot. Such “configural representation” does not require either a relational processing or a flexible use of the learned information [51], [52]. Although this hypothesis deserves further investigation, the qualitative differences in information processing between tasks suggest that neurogenesis could be more specifically involved in certain hippocampus-dependent processes.

The lack of impairment in contextual fear conditioning after inhibition of neurogenesis that we described here is consistent with some but not all previous studies [23]–[25]. Data concerning the water maze are more conflicting given that neurogenesis inhibition did not influence performances in this task in most studies [20], [22]–[25], while some deficits during the acquisition and/or the retention phase were found in others [18], [19]. Differences in the specificity of the methods used to ablate neurogenesis, in the age of the adult-born neurons that were depleted and in the degree of hippocampal-dependency of the studied tasks are potential explanations for these discrepancies [16].

The age of the newborn neurons that are depleted can explain the lack of effects found in some previous studies [19], [20], [24]. Indeed, in these reports neurogenesis was blocked between 2 and 4 weeks before behavioral testing, and the newborn neurons that are deleted thus seem too young to participate in learning tasks. This bias does not apply to our study since the water maze test started after at least six weeks of Dox treatment.

The age of newborn neurons cannot explain why an effect could not be observed in two recent studies in which neurogenesis was blocked three months before the start of the behavioral tests [21], [23]. In the first study, training was short (5 days) and control animals did not learn the task as indicated by the learning curve and the time spent in the target quadrant during the probe test. Indeed, the number of crossings of the location previously containing the platform (≈1,5, see Figure 3f in Meshi et coll.) is quite low compared to what we observed in our experiments (≈5, see Supplementary Figure S4K). Thus, it is not surprising that irradiation did not induce memory deficits in this condition. In the study of Saxe et al., animals were trained for 10 days and showed good memory for platform location during the probe test. It is more difficult to explain the discrepancies between our study and the one of Saxe and colleagues given that the protocol they used was not detailed enough.

Discrepancies in fear conditioning results could also be explained when considering that this task can also be learned with different degrees of hippocampal-dependency, depending on the features of the environment and on the conditioning procedure. Unfortunately, the level of hippocampal dependency of the tasks used in previous experiments investigating the effects of neurogenesis ablation was not described. The degree of hippocampal dependency of the task used in our experiments was analyzed here and in a previous study [40]. Indeed, we show that inactivation of the dorsal hippocampus leads to a behavioral impairment of 35% in our fear conditioning procedure, in which impairment does not exceed 65% even when the dorsal hippocampus is completely lesioned [40]. Thus, it appears that adult-born neurons are not essential to acquire contextual fear conditioning with simple experimental conditions. The lack of a deficit reported here is consistent with the observation that mice lacking NMDA receptor 1 specifically in DG granule neurons can acquire contextual fear conditioning in simple but not in complex conditions [53]. Altogether, our observations indicate that neurogenesis influences spatial learning only for tasks that require a critical implication of the hippocampus such as spatial relational memory [54]–[58].

The influence of neurogenesis on spatial relational memory is in agreement with a series of previous correlative pieces of evidence. For example spatial learning: i) influences the rate of production and the number of surviving newborn neurons [11]–[15]; ii) is positively correlated to the rate of neurogenesis [9] and, iii) activates adult-born neurons [59]. Furthermore, adult-born neurons express electrophysiological properties that contribute to long-term potentiation (LTP) in the DG [23], [60], [61], a synaptic mechanism that sustains learning and memory [62]. Finally, LTP influences the rate of production and survival of adult-born granule neurons [63]. The implication of DG neurogenesis in spatial relational memory is also supported by spatial relational memory deficits in the water maze after specifically disrupting the DG with colchicine lesions [64]–[67]. Given that adult-born neurons have also been involved in memory by linking memories across time it can be proposed that depletion of adult born neurons could lead to deficits in temporal ordering of spatial information [68], [69]. It remains to be determined whether the DG itself sustains this role whilst this latter is classically attributed to the CA1 subfield of the hippocampus [70].

The precise mechanism through which adult-born neurons participate in spatial relational memory remains to be elucidated. It has been shown that the hippocampus is important for the expression of remote spatial memory in the water maze, which is required for animals to navigate through space [54]–[58]. Thus, based on this knowledge, it is reasonable to hypothesize that newborn neurons could contribute to this process either by storing memory indexes [71] or by acting as binding detector cells, which more specifically encode the relationships between items [72].

The DG has also been proposed to process and represent spatial information on the basis of conjunctive encoding of multiple sensory inputs and of spatial pattern separation [73], [74]. After integrating all sensory inputs, the DG encodes and separates similar spatial events from each other via an orthogonalization of sensory input information. This process allows creating distinct memory representations for sets of information that share a certain degree of similarity and thus reduces spatial interference. In agreement with this view, it has been recently shown that minimal changes of the spatial environment explored by rats result in a strong decorrelation of activity patterns among place-modulated granule cells in the DG [75]. Behaviorally, evidences based on lesional or genetic approach also indicate that the DG contributes to pattern separation of spatial information [53], [76]. In this context it has been recently proposed that the constant production of new neurons in the DG ensures that each new event is encoded uniquely and consequently allows the dissociation (separation) of similar patterns [68], [69]. Thus the deficits in the water maze that we observed with multiple start location might be interpreted to result from an impairment in the use of spatial pattern separation process. Indeed, in the classical version of the water maze task [43], the use of variable start points from trial to trial allows the animal to acquire different spatial representations associated to the variable point of view at the departure. Spatial interferences may arise between juxtaposed or overlapped spatial memory representations. Thus, an increase in the amount of such interferences associated to an inability to extract and separate efficiently spatial representations may account for behavioral deficits during navigation in this task (for discussion see [76]). However, future studies are needed to confirm a specific role for adult-born neurons in this process.

In conclusion, by using a novel inducible transgenic strategy that allows specific ablation of hippocampal nestin precursors, we showed that hippocampal neurogenesis highly influences hippocampal-dependent complex cognitive functions, such as spatial relational memory, which is required for the establishment of associations between multiple spatial cues necessary for the encoding and use of a spatial map.

## Materials and Methods

### Plasmid construction

To obtain the pBI-EYFPBax-TetO-ECFPBax construct, a three-step strategy was used. First, in order to homogenize both polyA signals, we replaced the fragment containing the SV40 polyA signal from the pBI Tet vector (#6152-1, Clontech, USA) by the β-Globin polyA signal by PCR using Pfu DNA polymerase (Stratagene, USA) to minimize errors. The β-Globin polyA signal was amplified by PCR using the following oligonucleotides:

Forward primer: 5′ ACGCGTCGACGACTGAGAACTTCAGGGTGAGTTTGG 3′/Reverse primer: 5′ CTTTGACCAGCGTCATGCAGTCGAGTTCATAAGAGAAGAGG 3′. A Sal I restriction site in 5′ (underlined) and a PshAI restriction site in 3′ (underlined) were added in the forward and the reverse primer, respectively. Since Pfu DNA polymerase generates blunt ends, the PCR product was digested only by SalI and cloned by SalI/Blunt into the pBI Tet vector previously digested by SalI and NaeI (Blunt). The second and the third steps consisted of cloning the EYFPBax and ECFPBax cDNA. They correspond to fusion cDNAs in which the mouse Bax cDNA (GenBank accession number L22472) was digested at the 5′end by HindIII and at the 3′end by EcoRI and cloned into the 3′end of either the pEYFP-C1 vector (#6005-1, Clontech, USA) or the pECFP-C1 vector (#6076-1, Clontech, USA), respectively digested by HindIII/EcoRI. Indeed, the second step consisted of digesting the EYFPBax cDNA by NheI/SmaI enzymes from the pEYFP-C1 vector (Clontech, USA) followed by a Klenow blunting and cloning of the fragment into the pBI-Tet vector from the first step previously digested by SalI (blunted). Finally, the third step consisted of cloning the ECFPBax cDNA into the previous vector. The ECFPBax cDNA was digested by NheI/SmaI enzymes and cloned sticky/blunt into the previous vector digested by NheI/EcoRV. The resulting inducible construct, named pBI-EYFPBax-TetO-ECFPBax, expresses two genes (EYFPBax and ECFPBax) from one bidirectional Tet-responsive promoter.

### *In vitro* experiment

For *in vitro* transfection, we used a CHO-K1 Tet-On (#C3021-1, Clontech, USA) cell line which was stably transfected with the reverse tetracycline-controlled transactivator (rtTA) driven by the cytomegalovirus promoter. 24 hours post-transfection of the pBI-EYFPBax-TetO-ECFPBax vector, CHO-K1 Tet-On cells (Clontech, USA) grown on PolyLysine (Sigma)-coated coverslips were treated with doxycycline (Dox, 2 µg/ml; #D9891, Sigma, USA), a tetracycline analogue, for 6 hours. Then, the cells were fixed with 4% paraformaldehyde (10 min at room temperature), mounted in Mowiol (Calbiochem) and analyzed using a Zeiss LSM 510 META confocal microscope.

### Fluorescence Resonance Energy Transfer

FRET analysis of Bax multimers was used to track the intermolecular interaction between ECFPBax and EYFPBax fusion proteins. For cultured CHO cells, FRET was quantified using “Microfret” [77]. In brief, this method consists in the acquisition of three images through i) a CFP filter set (excitation 436/10 nm, emission 470/30 nm); ii) a YFP filter set (excitation 500/20 nm, emission 535/30 nm); iii) a FRET filter set (excitation 436/10 nm, emission 535/30 nm), and the determination of the “corrected FRET” or FRETc value which takes into consideration the non-FRET signal caused by the overlapping of the CFP and YFP spectra. Thus, the calculation of FRETc depends on the following relationship: FRETc = IFRET–CCFP–CYFP, where IFRET corresponds to the intensity of FRET with the FRET filter set of CFP/YFP-coexpressing cells; CCFP and CYFP correspond respectively to the crosstalk between CFP and YFP due to overlapping spectra. This phenomenon was evaluated in cells expressing only one of the two constructs, because the contribution of CFP and YFP to FRET in coexpressed cells is proportional to that in singly expressed cells. The right side of the equation was experimentally determined and our measurements revealed that 58.8±0.5% of CFP and 55.1±0.8% of YFP fluorescence can bleed through the FRET channel (average of 50 cells). Consequently we deduced that CCFP = 58.8%×ICFP and CYFP = 55.1%×IYFP, where ICFP corresponds to the intensity of CFP under the CFP filter set in coexpressing cells, and IYFP is the intensity of YFP observed under the YFP filter set in coexpressing cells. Finally, the calculation of FRETc corresponds to the following equation: FRETc = IFRET–(58.8%×ICFP)–(55.1%×IYFP). In addition, another measure of FRET, called FRETN was calculated. It corresponds to the ratio of FRETc by the mean fluorescence intensity of CFP and/or YFP and it allowed us to compare FRETc intensity between cells expressing different levels of CFP and YFP, as the FRETc value depends on the concentration of the present fluorophores: FRETcN = FRETc/(ICFP * IYFP). For each acquisition channel, images were analyzed using Metamorph 4.5 software (Universal Imaging Corporation).

*In vivo*, FRET efficiency was measured in areas devoid of (Ctrl) or containing (Bax) Bax multimers, by the increase of donor emission (ECFPBax) after quenching the acceptor emission (EYFPBax) by photobleaching. EYFP photobleaching was performed by repeated excitation with a 514 nm laser at 100% power. FRET efficiency was quantified on a Zeiss LSM 510 META confocal microscope using the dedicated LSM FRET module of the Zeiss AIM Software.

### Gene targeting

pBI-EYFPBax-TetO-ECFPBax was excised from the plasmid backbone by PshAI/AseI digestion. Microinjection into fertilized (C57BL/6JxCBA) F_2_ oocytes and other surgical procedures were performed as described earlier [78].

### Genotyping

Genomic DNA was isolated from tail clips and genotype determined using different sets of primers to discriminate between wild-type, monogenic heterozygous Nestin-rtTA, Bax and bigenic rtTA/Bax mice. The PCR protocols using Taq Polymerase (Biolabs, UK) to analyze rtTA and Bax transgenes, respectively, were 95°C 1 min, then 35 cycles of 95°C 45 sec, 56°C 45 sec, 72°C 2 min, then 72°C 10 min; and 95°C 1 min, then 30 cycles of 95°C 45 sec, 65°C 45 sec, 72°C 3 min 30 sec, then 72°C 10 min.

### Animals

Both Bax founder mice and mice expressing the transgene for the reverse tetracycline transactivator (rtTA) under the control of the rat nestin intron II enhancer/promoter [34] were amplified in a C57BL/6J (Charles River, Lyon, France) genetic background. From breeding of rtTA and Bax mice, 25% of bigenic mice, 25% of Bax mice, 25% of rtTA mice and 25% of wild-type mice were obtained. After weaning, male and female mice were separated and were housed 4–5 per cage until 2 months of age. Then, male mice were housed individually at the beginning of the Dox treatment and for the remainder of the experiments. A 12 hr light/dark cycle (lights on from 8:00–20:00) was used in the animal house. Temperature (22±1°C) and humidity (60±5%) were also controlled. All of the experiments were conducted in strict compliance with European Convention and institutional regulations.

### *In vivo* doxycycline treatment

Dox was administered through the drinking solution, supplemented with 2.5% sucrose. Dox-free animals were given 2.5% sucrose in the drinking solution (Vehicle). In experiment 1, male bigenic mice were treated for three weeks with the following doses of Dox: 0 mg/ml (n = 3), 1 mg/ml (n = 5), 2 mg/ml (n = 4), 4 mg/ml (n = 3). In an additional experiment, the inducibility of the Bax transgene was studied by sacrificing bigenic mice 0 min (n = 3), 2h (n = 4), 24h (n = 4) and 48h (n = 4) after an acute injection of Dox solution (2 mg/ml/100g body weight, i.p). For behavioral experiments (2^nd^ and 3^rd^ experiments), a dose of 2 mg/ml of Dox was chosen because it was found to have maximal effects on neurogenesis in experiment 1. Animals were treated with Dox for seventeen weeks (experiment 2) or nineteen weeks (experiment 3). For experiment 2, fifty mice were used: wild-type-vehicle = 7, rtTA-vehicle = 7, Bax-vehicle = 7, bigenic-vehicle = 6, wild-type-Dox = 6, rtTA-Dox = 4, Bax-Dox = 7, bigenic-Dox = 6. At the end of the experiment, we verified that bigenic-Dox mice did not differ from the control groups for body weight value (interaction genotype×treatment, F_1,46_ = 0.032, p>0.85) or for brain weight value (interaction genotype×treatment, F_1,46_ = 0.06, p>0.80). For experiment 3, fifteen mice were used: control littermates (wild-type, rtTA, Bax)-Dox (n = 7) and Bigenic-Dox (n = 8).

### 5-bromo-2′-deoxyuridine (BrdU) injections

BrdU (Sigma, dissolved in saline solution), a thymidine analogue incorporated into genetic material during the S phase of the cell cycle, was injected in order to label dividing cells. In the dose-response experiment (experiment 1), mice received two injections of BrdU (1×50 mg/kg/day) 11–12 days before sacrifice. In experiment 3, animals were injected with BrdU (1×100 mg/kg) and were allowed to survive for 4 additional weeks.

### Behavioral analysis

At 14 weeks of age, 6 weeks after the beginning of the Dox treatment, independent groups of mice were tested in two separate experiments (experiments 2 and 3) for three behavioral tasks, each task being separated by a one week interval. The order of the tasks in the two experiments was changed in order to control for the possible bias due to interactions between tasks or treatment duration (Figure 4). In experiment 2, novel environment exploration was tested twice, the first time at the beginning and the second time at the end of the behavioral sequence. Animals were then tested in a water maze followed by a fear conditioning test. In experiment 3 the opposite sequence was used. During water maze testing, in experiment 2 animals were first trained with a variable starting-point procedure, followed by a probe test, a visible platform test, and then by a constant starting point procedure before finally testing them with a novel start point. In experiment 3 the inverse order of water maze paradigms was used: animals were first trained with a constant starting-point procedure followed by testing with a novel starting point, and then a variable starting point procedure followed by a probe test, and finally a visible platform test.

#### Water maze

The apparatus was a white circular swimming pool (150 cm in diameter) located in a room with various distal cues. The pool was filled with water maintained at 20°C and made opaque by the addition of a non-toxic white cosmetic adjuvant. The escape platform (14 cm diameter) could be placed in different locations within the pool, raised above the surface of the water or hidden so that its top surface was 0.5 cm below the surface of the water. Data were collected using a video camera fixed to the ceiling of the room and connected to a computerized tracking system (Videotrack, Viewpoint) located in an adjacent room that also contained the individual home cages of the mice during testing. The tracking system allowed the calculation of escape latency (time required to find the platform, in seconds) and path length (distance covered by the mouse until it finds the platform, in centimeters).

#### Pre-training

Mice received a three-step pre-training session. First, mice were allowed to swim for 60 sec in the water maze without a platform. Then, they were placed upon the platform raised at the surface of the water where they were required to stay at least 15 sec. Finally, they were allowed to swim for a 30 sec period that was ended by a climbing trial onto the hidden platform. The platform was never localized in the quadrant used for the training sessions. At the end of the pre-training, all mice swam actively and were able to climb onto the platform and stay on it for 15 sec.

#### Training with variable start positions

In this task, mice were required to locate the hidden platform using distal extra-maze cues. For the entire training period they were tested with variable random start positions. They received 3 daily trials separated by a 5 minute inter-trial interval. During this period the cages were placed beneath a heat lamp to reduce core temperature loss. A trial terminated when the animal climbed onto the platform. Mice that failed to find the platform within a 60 second cut-off time were placed onto the platform by the experimenter and had to stay there for 15 sec before being placed back in their home cage for the 5 min inter-trial interval. The releasing point (starting point) differed for each trial and different sequences of releasing points were used day to day. Upon completion of the training, the hidden platform was removed and the memory for the platform location was assessed during a probe test. During this test mice were allowed to freely swim in the water maze for 60 seconds and performances were assessed by time spent in the target quadrant where the platform was located, and by total number of crossings in the exact platform location. Finally, on the last day, visual acuity and motor functions were tested by analyzing the ability of mice to reach a visible platform located in the quadrant opposite to the one that had contained the hidden platform.

#### Training with constant start positions

During this test mice were also required to locate the hidden platform using distal extra-maze cues. Procedures were similar to the ones used for training with variable start positions (3 daily trials with a 60 seconds cut-off and 5 min inter-trial intervals). However this time the start position was maintained constant between trials and days of training. Also the hidden platform was localized in a different quadrant than the one used for the variable-start paradigm. When performances reached a criterion of less than 10 sec of escape latency over two successive trials (2^nd^ day of training in experiment 2; 8^th^ day of training in experiment 3), animals were tested to locate the hidden platform from a novel start position that had never been used before (1 trial with a 60 seconds cut-off).

#### Locomotor activity

Locomotor activity was recorded in racks of 8 activity cages (18.2 cm×12 cm×22 cm) made of transparent Plexiglas and isolated from the surrounding environment. Each cage was equipped with two beams of infra-red captors located 1 and 8 cm from the floor, allowing recordings of horizontal activity and rearing, respectively. Infrared counts were computed via an electronic interface coupling each cage with an on-line computer (Imetronic, Bordeaux, France). Simultaneous recording of locomotor activity from sixteen animals took place from 2 to 4 pm under dim light (50 lux).

#### Contextual fear conditioning

Conditioning took place in a transparent Plexiglas box (30×24×22 cm high) with a floor made of 60 stainless steel rods (2 mm diameter, spaced 5 mm apart) connected to a shock generator (Imetronic, Bordeaux, France). The whole box was cleaned with 70% ethanol before each trial. For acquisition, all animals were submitted to a contextual conditioning consisting of a tone (CS)-shock (US) unpairing [79]. The animal was placed in the conditioning chamber for 4 min during which it received 2 foot shocks (0.7 mA, 50 Hz, 3 s), which never co-occurred with 2 tones (63 db, 1 KHz, 15 s). 24 hours later, mice were tested for their conditioned response using two retention tests during which their behavior was continuously video-taped for off-line scoring of freezing as an index of conditioned fear. Mice were first exposed to a neutral context (4 min, “Neut.Ctx”) and their conditioned fear responses were measured as the percentage of the total time spent freezing before (first two min) and during (2 min) the presentation of the tone CS. 2 hours later, mice were re-exposed to the conditioning context alone (6 min, “Cond.Ctx”) and their conditioned fear response, was measured as the percentage of the total time spent freezing during the first 4 min-period.

#### Hippocampal inactivation

In order to verify the hippocampal dependency of our fear conditioning procedure, supplementary groups of C57BL/6J male animals (n = 13) were implanted with bilateral hippocampal cannulae, and were injected with either lidocaine or vehicle solutions just before the conditioning session. Stainless-steel guided cannulae (26 gauge, 8 mm in length) were implanted bilaterally 1 mm above the dorsal hippocampus (A/P, −2000 µm; M/L, ±1300 µm; D/V, 1000 µm). Guides were permanently fixed to the skull with dental cement and two jewel screws and were obturated by appropriate stylets until behavioral manipulations. After surgery, mice were allowed to recover for at least 2 weeks. For infusions, the stylets obtruding the guide cannulae were removed. Stainless-steel cannulae (32 gauge, 9 mm) attached to 1 µl Hamilton syringes (PolyLabo, Strasbourg, France) with polyethylene catheter tubing were inserted through the guide cannulae. The syringes were fixed in a constant rate infusion pump. Each mouse was given bilateral infusions of 0.9% saline (Vehicle, Veh, 0.3µl/side) or 4% Lidocaine (Lido, Sigma, 0.3µl/side) into the dorsal hippocampus. Injections were delivered 5 min before the acquisition of fear conditioning. The cannulae were left in place for an additional 3 min before removal to allow diffusion of the drug away from the cannulae tips.

### Immunohistochemistry

At the end of experiments, mice were perfused transcardially with a phosphate-buffered solution of 4% paraformaldehyde and brains were cut on a vibratome (40 µm thick sections). For each staining, one in ten free-floating sections was processed according to a standard immunohistochemical procedure [9]. HH3 immunoreactivity (IR), nestin-IR, activated caspase 3-IR, Dcx-IR, CD11b-IR or PECAM-1-IR were revealed using respectively a rabbit anti-HH3 polyclonal antibody (1∶2000, Upstate), a mouse anti-nestin monoclonal antibody (1∶1000; Chemicon), a rabbit anti-activated caspase 3 polyclonal antibody (1:500, Cell signaling technology), a sheep anti-Dcx polyclonal antibody (1∶2000; SantaCruz), a rat anti-mouse CD11b monoclonal antibody (1∶1000; Serotec) and a rat anti-mouse PECAM-1 monoclonal antibody (1:500; Pharmingen). For BrdU labeling, sections were treated with 2N HCl (30 min at 37°C) and then incubated with a rat monoclonal anti-BrdU antibody (1∶2000, Accurate, New York, USA). For each staining, sections were processed in parallel and immunoreactivities were visualized by the biotin-streptavidin technique (ABC kit, Dako) using 3,3′-diaminobenzidine as a chromogen. Sections were counterstained with thionine in order to visualize apoptotic (pyknotic and karyorhexic) cells.

### Stereological analysis

The number of X-IR cells was counted under a 100× microscope objective. The total number of X-IR cells in the granular and subgranular layers of the DG was estimated using a modified version of the optical fractionator method on a systematic random sampling of every tenth section along the rostro-caudal axis of the DG [14]. Results are expressed as the total number of X-IR cells in the entire DG. In the OB, the number of BrdU-IR cells was estimated using an optical fractionator method (Stereo Investigator software, Microbrightfield). For each one-in-ten section, BrdU-IR cells were counted in 40 µm×40 µm ×10 µm frames at evenly spaced x-y intervals of 200 µm by 200 µm.

### Cell density measurements

The X-IR cells densities were estimated by the number of X-IR cells divided by the area of the structure measured with the Stereo Investigator software (Microbrightfield). Results are expressed as a mean number of X-IR cells/mm^2^.

### Optical density measurement

Nestin-immunostaining was quantified by measuring optical density with the image analyzer Samba 2005 R (Alcatel, Grenoble, France). The area of the granular cell layer was determined and staining density was calculated by dividing the pixel count by the overall area (pixels per mm^2^) after subtraction of the background value [80], [81].

### Measurement of EYFPBax intracellular aggregates

EYFPBax clusters were visualized using a Zeiss LSM 510 META confocal microscope and counted individually in the SVZ-OB system, the dentate SGZ, the cerebellum, the striatum, and the frontal cortex. Results are expressed as number of aggregates per section.

### Analysis of cellular phenotypes

In order to examine the phenotype of EYFPBax cells, one in ten sections was incubated with a mouse monoclonal anti-NeuN antibody (1∶1000) or with a mouse anti-nestin monoclonal antibody (1∶1000). Bound anti-NeuN or anti-nestin antibodies were visualized with Cy3-labeled anti-mouse IgG antibodies (1∶1000). EYFPBax cells aggregates expressing NeuN or nestin were determined using a Zeiss confocal microscope.

### Statistical analysis

Differences between groups were analyzed using a Student's *t* test or an ANOVA which was followed by a *post hoc* comparison using the Newman-Keuls test when necessary.

## Supporting Information

Figure S1 Confocal illustrations of a long-term Dox treatment on Bax transgene expression. EYFPBax (green) cytoplasmic clusters (white arrows) were located in the Dentate Gyrus (DG) of bigenic-Dox mice but none in the olfactory bulb, the cerebellum, the striatum and the frontal cortex. Cells were counterstained with hoescht (blue). Scale bar = 20 μm.
(3.31 MB TIF)

Figure S2 Effect of the induction of Bax proteins on newborn cells along the rostro-caudal axis of the dentate gyrus.
(0.35 MB EPS)

Figure S3 *In vivo* individual genomic integration of EYFPBax-TetO-ECFPBax or Nestin-rtTA-βGeo constructs does not influence behavioral abilities and adult neurogenesis. Wild-type (Wt), Bax and rtTA mice treated or untreated with Dox were compared to Bigenic rtTA/Bax mice treated with vehicle (Big-Veh). (A) Number of dividing cells in the DG identified by HH3 staining (F_6,37_ = 0.48, p>0.82). (B) Number of immature neurons in the DG measured by Dcx staining (F_6,37_ = 0.34, p>0.90). (C) Exploration in a novel environment measured by photocell beam breaks (group effect: F_6,37_ = 0.18, p>0.97; interaction group×time: F_66,407_ = 0.78, p>0.88). (D) Percentage of freezing displayed by mice in basal condition (“Base”) or when re-exposed (for 4 min, “Cond.Ctx”) to the shock-associated context 24 hours later (group effect: F_6,74_ = 1.75, p>0.12; conditioning effect: F_1,74_ = 150.33, p<0.001; interaction group×conditioning: F_6,74_ = 0.72, p>0.63). (E) Acquisition of spatial memory measured by the latency for reaching the hidden platform in the water maze using variable start positions (V1-9, group effect: F_6,37_ = 0.22, p>0.96; group×time interaction: F_48,296_ = 0.82, p>0.80). (F) Acquisition of spatial memory in the water maze using constant start positions (C1,C2, group effect: F_6,37_ = 1.70, p>0.14; group×time interaction: F_6,37_ = 0.63, p>0.70) and performance to reach the platform using a novel start position (N, F_6,37_ = 1.75, p>0.13).
(0.24 MB EPS)

Figure S4 Cell death induced by the over-expression of Bax proteins does not cause a vascular response or mild inflammation in the adult dentate gyrus. (A) Densitometric analysis of PECAM-1-IR. (B) Illustrations of blood vessels visualized by PECAM-1 staining (platelet endothelial cell adhesion molecule-1). (C) Total number of CD11b-IR cells. (D) Illustration of microglia cells visualized by complement type 3 receptor CD11b staining. Inset: high magnification of a CD11b-IR microglia cell.
(0.41 MB EPS)

Figure S5 Complete set of behavioral results. *In the behavioral tasks of experiment 2*, bigenic rtTA/Bax mice treated with vehicle (BV) were compared with those treated with Dox (2mg/ml, BD). (A) Exploration in a novel environment measured by photocell beam breaks (group effect: F_1,10_ = 1.25, p>0.28; interaction group×time: F_11,110_ = 0.71, p>0.72). (B) Latency to reach the hidden platform using variable start positions (V1-9, group effect: F_1,10_ = 16.33, p<0.002; group×time interaction: F_8,80_ = 2.80, p<0.008). Right panel: swim path illustrations during the last training day. (C) Time spent in the target quadrant during the probe test (BV, t_5_ = 8.92, p<0.001; BD, t_5_ = −0.50, p>0.63 against chance level). Right panel: swim paths illustration. (D) Latency for reaching the hidden platform using constant start positions (C1, C2, group effect: F_1,10_ = 0.34, p>0.57; group×time interaction: F_1,10_ = 0.04, p>0.83) and a novel start position (N, t_10_ = −2.45, p<0.05 compared to C2; t_10_ = −2.61, p<0.05 compared to BV). Right panel: illustration of swim paths to reach the platform when the starting position was constant or novel. (E) Contextual fear conditioning assessed by the percentage of freezing displayed by mice before conditioning (Base) or when re-exposed to the conditioning context (Cond.Ctx) 24 hours later (Base *vs* Cond.Ctx: BV, t_10_ = −4.59, p<0.001, BD, t_10_ = −5.23, p<0.001; Cond.Ctx: BV *vs* BD: t_10_ = 0.56, p>0.60). (F) Exploration measured by photocell beams breaks at the end of the tasks sequence (group effect: F_1,10_ = 1.16, p>0.30; interaction group×time: F_11,110_ = 0.26, p>0.99). *In the behavioral tasks of experiment 3*, control mice (Wt, Bax, rtTA = CD) and bigenic mice (BD) treated with Dox (2mg/ml) were compared. (G) Fear conditioning assessed by the percentage of freezing displayed 24 hours after conditioning by mice exposed to a neutral context (Neut.Ctx) before (Pre-tone) and during (Tone) re-exposure to the tone previously presented unpaired to the shock during conditioning (Pre-tone *vs* Tone: CD, t_12_ = −0.32, p>0.75, BD, t_14_ = −0.48, p>0.63). (H) Contextual fear conditioning assessed by the percentage of freezing displayed by mice before conditioning (Base) or when re-exposed to the conditioning context (Cond.Ctx) 24 hours later (Base *vs* Cond.Ctx: CD, t_12_ = −4.34, p<0.001, BD, t_14_ = −9.26, p<0.001; Cond.Ctx: CD *vs* BD, t_13_ = −1.26, p>0.23). (I) Latency to reach the hidden platform using a constant start position (C1-8, group×time interaction: F_7,91_ = 1.41, p>0.21; C8, t_13_ = −0.055, p>0.58) or a novel start position (N, t_14_ = −6.17, p<0.001 compared to C8; t_13_ = −6.24, p<0.001 compared to CD). Right panel = swim path illustrations with constant or novel start positions. (J) Latency to reach the hidden platform using variable start positions (V1–4, group effect: F_1,13_ = 21.32, p<0.001; group×time interaction: F_3,39_ = 1.34, p>0.27). Right panel: swim path illustrations during the last training day. (K) Time spent in the target quadrant (CD: t_6_ = 9.24, p<0.001 and BD: t_7_ = −0.80, p>0.45 against chance level) and total number of crossings within the platform location during the probe test (t_13_ = 4.39, p<0.001). Right panel: swim paths illustration. *: p≤0.05, **: p≤0.01, ***: p≤0.001 compared to the control group; #: p≤0.05 compared to C2, ###: p≤0.001 compared to C8; +++: p≤0.001 compared to chance level. °°°: compared to Base.
(0.74 MB EPS)

Table S1 Effects of a long-term Dox treatment on Bax transgene expression. The EYFPBax cell density and the caspase 3-IR cell density were estimated in the olfactory bulb, the cerebellum, the striatum and the frontal cortex after long-term Dox treatment in bigenic-Dox mice and compared to bigenic-Vehicle mice.
(0.42 MB EPS)

Table S2 *In vivo* expression of Bax transgenes does not influence cell proliferation in the subventricular zone.
(0.40 MB EPS)

Table S3 *In vivo* expression of Bax transgenes does not affect granule cell layer parameters.
(0.40 MB EPS)

Table S4 Influence of adult hippocampal neurogenesis ablation on spatial navigation-related parameters. (A) The positive correlation between latency to reach the hidden platform and swim path length during the last training session using multiple start departure (Figure 6A, “V9”) indicates that the behavioral deficit of bigenic-Dox mice does not rely on a non-cognitive strategy such as “passive floating”. (B) The total swim path length and average swim speed during the probe test indicates that bigenic-Dox mice do not possess disrupted motor abilities. In addition, the estimation of the time spent swimming near the walls of the water maze indicates that bigenic-Dox mice are not inclined to use another non-cognitive swim strategy such as “thygmotaxis”. (C) Latency, swim path and average speed to reach a visible platform during the cued test further indicate that the inability to locate a hidden platform of bigenic-Dox mice is not related to visual acuity deficits or altered general health status.
(0.42 MB EPS)
