# Supplementary figures and images for: Spatial Relational Memory Requires Hippocampal Adult Neurogenesis
Source: PLoS One. 2008 Apr 9;3(4):e1959. doi: 10.1371/journal.pone.0001959 (PMC2396793; doi:10.1371/journal.pone.0001959)

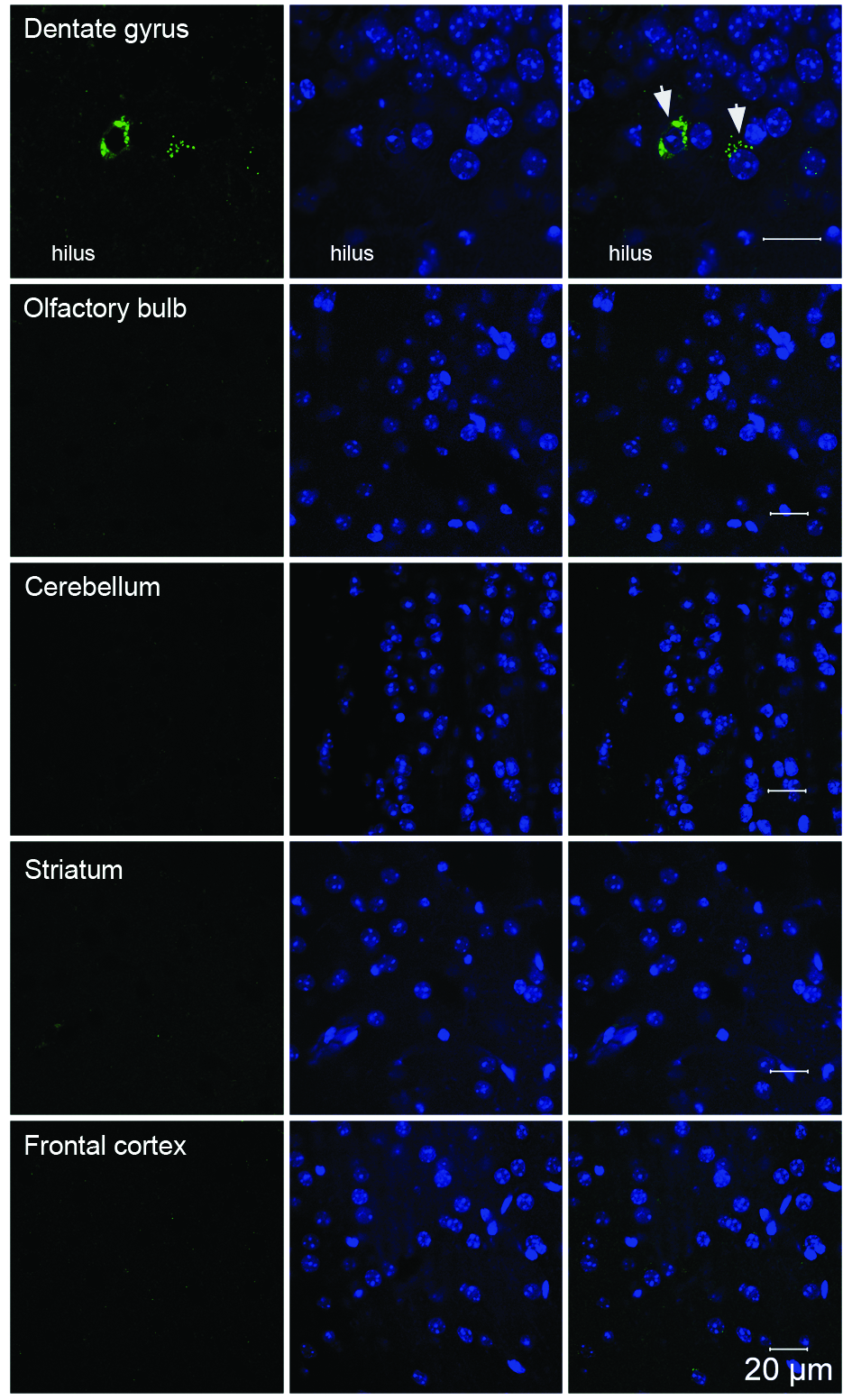

Supplement: Figure S1 — Confocal illustrations of a long-term Dox treatment on Bax transgene expression. EYFPBax (green) cytoplasmic clusters (white arrows) were located in the Dentate Gyrus (DG) of bigenic-Dox mice but none in the olfactory bulb, the cerebellum, the striatum and the frontal cortex. Cells were counterstained with hoescht (blue). Scale bar = 20 μm. (3.31 MB TIF) [file pone.0001959.s001.tif]
